# Association of neutrophil gelatinase-associated lipocalin (NGAL) and blood pressure in children with Henoch Schönlein Purpura

**DOI:** 10.1186/1546-0096-10-S1-A124

**Published:** 2012-07-13

**Authors:** Pamela Weiss, Andrew J Klink, Kevin Meyers, Russell Localio, Mary B Leonard, Chris Feudtner

**Affiliations:** 1 Children's Hospital of Philadelphia, Philadelphia, PA, USA; 2 University of Pennsylvania, Philadelphia, PA, USA

## Purpose

Renal disease often occurs with Henoch Schönlein Purpura (HSP). NGAL is a non-invasive, urinary biomarker that is predictive of renal flares in SLE1 and associated with elevated SBP and DBP in subjects with preeclampsia2 or early atherosclerosis3. We hypothesized that elevated NGAL is associated with elevated BP and proteinuria in children with HSP.

## Methods

We conducted a 6-month prospective cohort study of subjects <18 years who were evaluated for new-onset HSP between February 2009 and June 2010. Weight, height, and casual BP were recorded at 0-7, 60 and 180 days and first-morning urine samples were collected at 0-7, 14, 28, 60, and 180 days after diagnosis. Twenty-four-hour ABPM was completed 6 months after diagnosis. BP measurements were standardized using body mass index and BP Z-scores (casual) and BP index (ABPM). Mixed effects regression models with pre-specified individual level covariates (age and gender) as fixed effects and subject as a random effect were used to examine the relationship between urinary NGAL and the following: 1) casual SBP and DBP over time, 2) ABPM at 6 months after diagnosis, and 3) urine protein to creatinine ratio over time.

## Results

21 subjects with new-onset HSP were enrolled in the cohort. Median age was 5 years (IQR: 4, 8). Fifty-two percent of subjects were male. Sixty-three percent and 16% had a urine protein to creatinine ratio >0.2 and >0.5, respectively, during the study. Thirteen percent and 13% had 24-hour SBP or DBP >95th% on ABPM. Thirty-six percent, 7%, and 36% had abnormal systolic, diastolic, and MAP nocturnal dipping on ABPM, respectively. Thirty-seven percent and 26% had standardized casual SBP and DBP >95th% during the study. Urinary NGAL on the log-scale was not significantly associated with casual SBP over time, ABPM SBP or DBP, or the urine protein to creatinine ratio. Increased urinary NGAL levels on the log-scale were significantly associated with increased casual DBP over time (coefficient: 3.7, 95% CI: 1.8, 5.7) and abnormal nocturnal systolic dipping (coefficient -1.68, 95% CI: -3.05, -0.32). Increased urinary NGAL was also associated with abnormal nocturnal diastolic and MAP dipping, albeit statistically insignificant.

## Conclusion

In this pilot investigation of the relationship between urinary NGAL and BP and proteinuria in children with HSP, in a relative small sample of patients we found a significant association between 1) urinary NGAL and DBP over time, and 2) urinary NGAL and abnormal nocturnal dipping using 24-hour ABPM 6 months after diagnosis. Larger investigations and investigations over more time are needed to better define the relationship of NGAL with both BP and other renal outcomes in children with HSP.

## Disclosure

Pamela Weiss: None; Andrew J. Klink: None; Kevin Meyers: None; Russell Localio: None; Mary B. Leonard: None; Chris Feudtner: None.

